# A Machine Learning Enhanced Mechanistic Simulation Framework for Functional Deficit Prediction in TBI

**DOI:** 10.3389/fbioe.2021.587082

**Published:** 2021-03-03

**Authors:** Anna Schroder, Tim Lawrence, Natalie Voets, Daniel Garcia-Gonzalez, Mike Jones, Jose-Maria Peña, Antoine Jerusalem

**Affiliations:** ^1^Department of Engineering Science, University of Oxford, Oxford, United Kingdom; ^2^Nuffield Department of Clinical Neurosciences, University of Oxford, Oxford, United Kingdom; ^3^Institute of Medical Engineering and Medical Physics, Cardiff University, Cardiff, United Kingdom; ^4^Lurtis Ltd., Oxford, United Kingdom

**Keywords:** traumatic brain injury, resting state functional magnetic resonance imaging, default mode network, finite element simulation, machine learning

## Abstract

Resting state functional magnetic resonance imaging (rsfMRI), and the underlying brain networks identified with it, have recently appeared as a promising avenue for the evaluation of functional deficits without the need for active patient participation. We hypothesize here that such alteration can be inferred from tissue damage within the network. From an engineering perspective, the numerical prediction of tissue mechanical damage following an impact remains computationally expensive. To this end, we propose a numerical framework aimed at predicting resting state network disruption for an arbitrary head impact, as described by the head velocity, location and angle of impact, and impactor shape. The proposed method uses a library of precalculated cases leveraged by a machine learning layer for efficient and quick prediction. The accuracy of the machine learning layer is illustrated with a dummy fall case, where the machine learning prediction is shown to closely match the full simulation results. The resulting framework is finally tested against the rsfMRI data of nine TBI patients scanned within 24 h of injury, for which paramedical information was used to reconstruct *in silico* the accident. While more clinical data are required for full validation, this approach opens the door to (i) on-the-fly prediction of rsfMRI alterations, readily measurable on clinical premises from paramedical data, and (ii) reverse-engineered accident reconstruction through rsfMRI measurements.

## 1. Introduction

Traumatic brain injury (TBI) is one of the leading causes of death in people under the age of 45 years (Maas et al., 2008). In the EU, it is estimated that 2.5 million people suffer annually from TBI (Maas et al., 2015). While they can also result from non-impact conditions such as blast waves arising from an explosion, most TBIs occur as a consequence of head impacts, e.g., during falls, road traffic accidents, assaults, and sport injuries. The impact conditions can be very diverse, as expected from the large parameter space characterizing the boundary conditions of the contact (location, impact velocity, angle of impact, impactor shape, impactor material properties, etc.), as well as the high sensitivity associated to some of these (Fahlstedt et al., 2012). Despite improvements in care, functional outcomes are equally variable, even among those with apparently minor early injury severity. The limited predictive power of current clinical head injury scales raises a prominent need for tools better able to anticipate the long-term effects of TBI.

To understand better the effects of these impact conditions, computational models, and, in particular, finite element head models (FEHMs), have been used to predict mechanical deformation and stress levels on brain tissue (Raul et al., 2008; Dixit and Liu, 2017). This approach has typically been leveraged to correlate mechanistic measures (e.g., pressure, von Mises stress, principal strains, etc.) with different degrees of tissue damage. Historically, FEHMs have successfully been utilized for the prediction of structural events such as skull fracture (Garcia-Gonzalez et al., 2017). However, as local mechanical disturbances in the brain can lead to time-dependent systemic biological and multiphysics responses, these models are intrinsically unable to mechanistically predict functional alterations or cognitive deficits. Barring a few exceptions (Garcia-Gonzalez et al., 2018b), very little work has focussed on correlating functional deficits, tissue damage, and mechanical features in a fully validated framework, e.g., with clinical or animal data. Even then, in most of the cases, the high cost (both in man-hour and computational) to develop, run, and analyze the underlying FEHM remains extremely impractical and not fit for direct clinical use. While coupling mechanistic approaches to machine learning (ML) methods has been recently highlighted as a potential avenue for alleviating these restrictions (Baker et al., 2018), very little has been done in this field.

At the clinical end of the spectrum, the diagnosis and prognosis of TBI rely heavily on the clinician's experience. Indeed, while a lot of effort has focused on outcome prediction—outcome being often defined in relatively broad terms, e.g., “mortality” or “unfavorable outcome” (Roozenbeek et al., 2012)—, these prognostic models are not directly usable for individual patients (Menon and Harrison, 2008). Instead, head injury assessment by healthcare professionals still relies on general guidance built around a set of recommendations such as the ones provided by the National Institute for Health and Care Excellence (National Institute for Health and Care Excellence, 2019). Even then, the immediate cognitive evaluation of the sufferer is generally based on the Glasgow Coma Scale (GCS) originally defined in mid 70s (Royal College of Physicians and Surgeons of Glasgow, 1974), and solely focused on symptoms as opposed to cause identification.

The recent development in magnetic resonance imaging (MRI) has allowed for the identification of new candidates for direct functional evaluation of the brain. In particular, resting state functional MRI (rsfMRI) is a technique that identifies correlated networks in the absence of specific tasks (Fox and Raichle, 2007), offering insight into network function among unconscious patients unable to engage in active cognitive tasks (Kondziella et al., 2016). Among the common findings in patients with TBI is the alteration to the default mode network (DMN) (Sharp et al., 2014). While rsfMRI could hold the key to a more direct and straightforward diagnosis of eventual cognitive deficits in TBI, a prognostic/diagnostic tool to link network alteration and tissue damage still remains elusive.

To this end, this work proposes a new method aimed at predicting rsfMRI network deficit directly from trauma data by means of a ML layer taking as inputs a combination of impact conditions, namely: location, velocity of impact, angle of impact (represented by a binary input indicating whether the impact is perpendicular or not), and shape of the impactor (represented by their radii of curvature). The ML layer predicts the extent of tissue damage after being trained by a library of pre-simulated impact loaded FEHMs for which a shear energy rate threshold is used to estimate the percentage of tissue damage in the DMN. Our results show that it is able to capture very well the proportion of brain damage sustained mechanically, and thus alleviate significantly the computational time experienced by direct FEHM simulations. In parallel to this, a functional criterion defined as the proportion of brain voxels statistically decoupled from the neurologically normal DMN is proposed to quantify the functional damage to the DMN. Both mechanistic and functional criteria are then evaluated for nine TBI patients with clinical and rsfMRI data available in the hyper-acute phase (first few hours) after trauma, for whom the accident is reproduced *in silico* from paramedical data. Despite a very wide variability in the extent of the predicted DMN tissue damage, the mechanical damage values are generally aligned in trend with the “ground truth” functional damage observed in these patients as quantified by the functional criterion. Assuming a direct relationship between the two criteria, the proposed framework is ultimately used to estimate the real velocity of impact experienced by the nine patients.

While future validation work is needed to extend these model predictions to an even more comprehensive range of head injuries, we propose that this virtual prediction framework offers avenues for realistic estimation of either brain functional deficit when knowing the accident conditions, or the accident conditions when having access to the functional evaluation. Such estimations have direct clinical utility in the general clinical setting where very rare hyper-acute MRI scans, used to validate model predictions here, are not obtainable. Once fully validated on a larger cohort, this approach could find a direct use in clinical and forensic environments.

## 2. Materials and Methods

### 2.1. Clinical Data

#### 2.1.1. Participants

Adult patients (aged 18 years and over) were prospectively recruited from the Emergency Department at the Oxford University Hospitals NHS Foundation Trust as early as possible following traumatic head injury. Eighteen patients in total were recruited, among which nine patients (mean age: 55.8 range: 22–83) were selected for this study based on having a single defined mechanism of injury suited to modeling. Patients underwent a CT scan as part of standard trauma care. Once immediately life-threatening conditions were identified and treated, patients were recruited for a research MRI scan within 24 h of injury (the “hyper-acute” phase). Patients who were intubated and ventilated at the time of recruitment were transferred to MRI by a dedicated neuro-intensive care team consisting of a consultant neuro-anaesthetist, neuro-intensive care nurse, MRI research nurse, and consultant neurosurgeon. The same team managed the patient throughout the scan before transferring to intensive care. Patients were excluded from the study if they had contraindications to MRI, injuries requiring urgent surgery, or were medically unstable so that scanning would not be safe. Patients were followed throughout their hospital stay and returned at 6–9 months following injury for repeat assessments. Initial severity of injury was assessed using post-resuscitation GCS at presentation. Severity, at 1 week/short term outcome, was assessed using both the GCS and location of the patient (in hospital or discharged). Patients with a GCS of 12 or less were considered moderate-severe. Patients with a GCS 13–15 were considered mild. All patients still in hospital with a GCS 12 or less and/or still in hospital (due to TBI) at 7 days were considered moderate-severe at this point. A neurological examination and Glasgow Outcome Scale (extended)—GOSe—questionnaire were completed at 6–9 months by the patients or their relatives/carers if they were unable. The mechanism of injury for every patient was ascertained from medical notes recorded at the scene or in discussion with the patient/witnesses. Patient demographics and clinical data are presented in Table 1.

**Table 1:** GCS, Glasgow Coma Scale; GOSe, Glasgow Outcome Score (extended).

**Case**	**Age**	**Sex**	**Mechanism of injury**	**Severity at presentation**	**GCS (15-point scale), at 7–9 days**	**GOSe at 6–9 months (8-point scale)**
1	83	M	Fall from a 2-story house, injuries suggestive of hip and left frontal head impact	Severe	Intubated and sedated	3
2	22	M	Closed hand hit to the face, fell backwards	Moderate	15	7
3	70	F	Kicked by a horse in the abdomen and head, fell backwards	Mild	15	8
4	30	M	Pedestrian, hit by van traveling at 20–30 mph. Hit head on wing mirror and knocked to the floor	Mild	15	8
5	61	F	Pedestrian, hit by cyclist. Impact to right side of the head behind ear	Moderate	15	4
6	52	F	Pedestrian, hit by car traveling at approx. 30 mph. Impact to right orbital/frontal region of the head	Moderate	15	8
7	41	M	Fall from 3-story roof. Impact to left temporal and frontal head regions. Additional right wrist fracture, cervical, and thoracic transverse spinous process fractures	Severe	Intubated and sedated	5
8	62	M	Charge by bull running out of a cattle truck. Thrown back against a metal fence and onto concrete. Landed on back of head	Moderate	15	6
9	72	M	Cyclist, knocked off bike, landed on head. Impact to left parietal head region and left shoulder.	Severe	Intubated and sedated	3

*The GOSe provides a numeric measure corresponding to the degree of disability a patient is left with after a head injury (Weir et al., 2012); lower scores indicate worse outcomes. GOSe differs from the GCS which is a 15-point measure of a patient's degree of consciousness, typically used as a marker of severity following injury (lower scores indicate more severe injury). The scale is intended for use after discharge from hospital, and in particular, moderate disability and good recovery are not assessable until after discharge. Assessment beyond 6 months is considered to provide a reasonable marker of the long-term cognitive effects attributable to the TBI (Dikmen et al., 2009)*.

Eighteen healthy controls, age and sex-matched to the patients, were recruited for normative data. Exclusion criteria for controls included contraindications to MRI and any current or historical neurological or psychiatric conditions. Healthy controls provided informed written consent. All patients with capacity at the time of initial recruitment gave written informed consent. For patients lacking capacity, the lead clinician, in consultation with the family, signed a declaration form to confirm agreement for the patient to be recruited into the study. Explicit patient consent was sought as soon as possible upon recovery. The study was approved by the South Central-Berkshire Research Ethics Committee.

#### 2.1.2. MRI Data Acquisition

MRI data were acquired on a 3T Siemens Magnetom Verio scanner at the Oxford Acute Vascular Imaging Centre (AVIC). The scanning protocol included T1-weighted MPRAGE and resting fMRI, acquired using an echo-planar T2*-weighted imaging sequence. The resting fMRI sequence parameters were: voxel size of 3 × 3 × 3 mm^3^, multiband acceleration factor: 2, repetition time: 1,640 ms, echo time: 30 ms, acquisition time: 05:35 min. Field maps were acquired to allow for correction of field inhomogeneity-induced geometric distortions in the fMRI data.

#### 2.1.3. rsfMRI Data Pre-processing

The rsfMRI data were analyzed using dedicated tools in the FMRIB Software Library (FSL) (http://fsl.fmrib.ox.ac.uk/fsl). First, standard pre-processing was performed, including brain extraction, motion correction, distortion correction using field maps, spatial smoothing (full-width at half maximum of 5 mm), and high-pass temporal filtering (100 s). To enable between-subject comparisons, individual subjects' functional scans were linearly registered to their respective high resolution structural (T1) scans and then nonlinearly aligned to the Montreal Neurological Institute (MNI) standard template brain, accounting for any gross brain pathology (such as contusions, haematoma).

**Figure 1:**
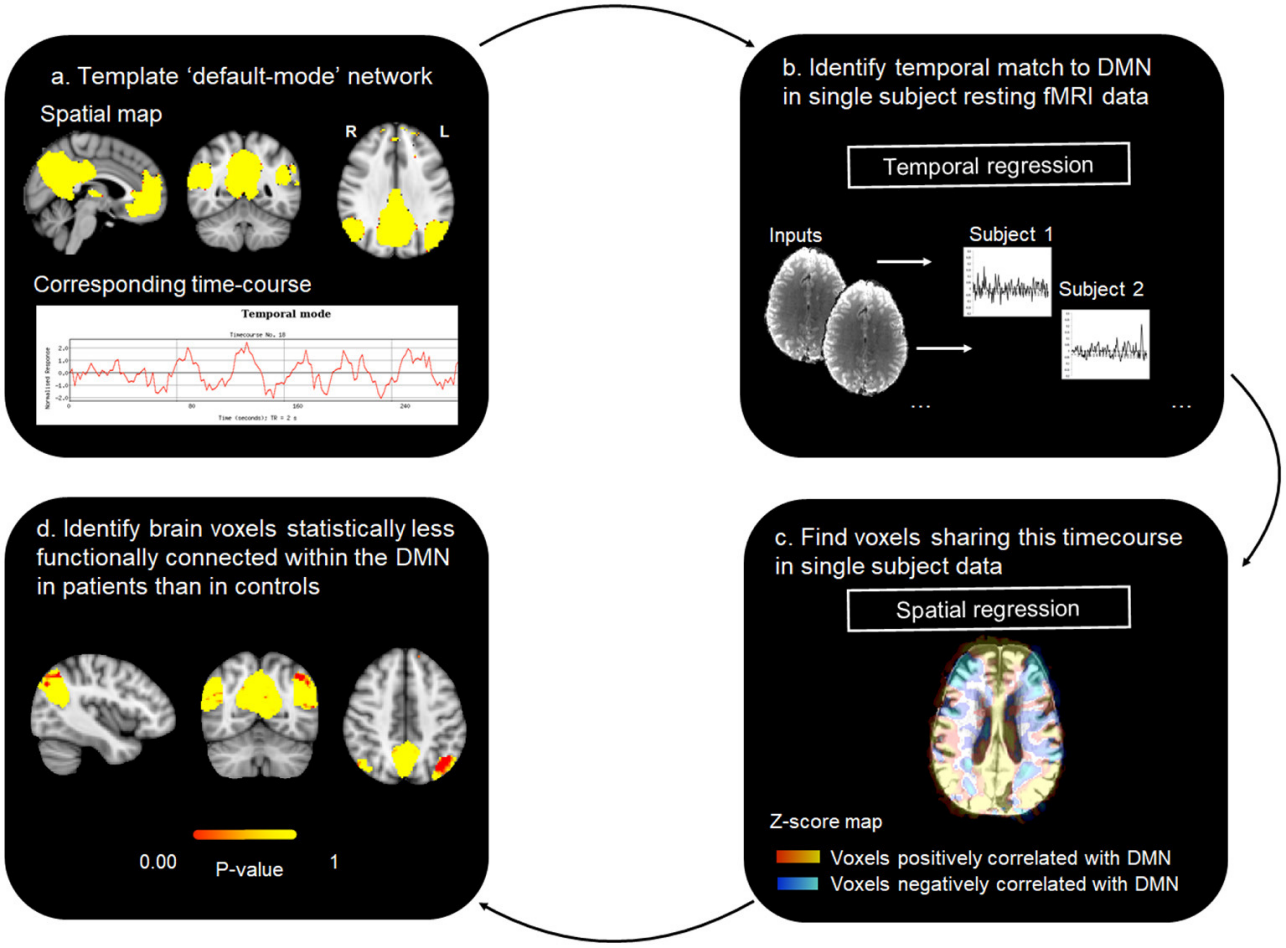
rsfMRI DMN analysis workflow.

Next, in order to objectively extract the DMN from each individual participant's resting fMRI data, we performed a dual-regression analysis, as previously described (Khalili-Mahani et al., 2012; Voets et al., 2012). For this analysis, we obtained a template set of 10 well-validated resting state networks (including the DMN) identified in healthy adults (Smith et al., 2009). A two-stage (temporal and spatial) regression was then performed. Each template resting network has a characteristic time-course. Therefore, in the first stage, each of the template networks was regressed against the rsfMRI time-series acquired in our individual subjects to identify time-courses corresponding to each template component (Voets et al., 2012). The second stage then identified brain voxels that shared this time course, for each of the 10 networks separately, from which we selected the DMN for further analysis. In this way, we obtained z-normalized single subject spatial maps, representing for every voxel in the brain the strength of its functional connectivity with the DMN in our nine patients and eighteen healthy controls (see Figure 1).

#### 2.1.4. Resting State Network Based Damage

Finally, we performed single-subject case-control statistical analyses. The objective of these analyses was to generate a DMN “damage load” index for every TBI patient by calculating the number of voxels in each patient's DMN whose connectivity was altered when compared to healthy controls. Since distributions in small samples may violate the assumptions underlying single case *t*-test analyses, for this analysis, we performed inference testing using Permutation Analysis of Linear Models (PALM) with signal flipping (Winkler et al., 2014), as described previously (Voets et al., 2017). These analyses were constrained to cortical voxels by constructing a group mean gray matter mask from automatic tissue segmentations of each subject's T1-weighted anatomical scan obtained using FSL-FAST (Zhang et al., 2001). For each patient, we compared the whole-brain DMN connectivity map to the distribution of connectivity maps generated from our 18 healthy controls using the general linear model framework. Two groups were created (corresponding to *n* = 18 healthy control and *n* = 1 patient, substituting the data for each of the nine patients in turn) and a single contrast (controls > patient, testing for voxels with lower DMN functional connectivity in the patient compared with controls). We performed 5,000 permutations for each case-control analysis and report permutation *p*-values for significant voxels using the thresholding method Cluster Free Threshold Enhancement (TFCE). TFCE offers a simple approach for calculating cluster-like voxel-wise statistics, providing sensitivity both to local maxima and spatial extent of signal without the need to define an arbitrary hard initial cluster-forming threshold (Smith and Nichols, 2009).

To obtain the individual patient DMN “damage load” metric, the resulting t-statistic maps (not corrected for multiple comparisons) were thresholded at a *p*-value of 0.05 to calculate the number of statistically “disconnected” voxels. Finally, the number of “disconnected” voxels was expressed as a percentage of the total DMN mask. The latter was calculated by generating a binary mask of the DMN, by thresholding the template DMN mask (Smith and Nichols, 2009) at z-score of 3.1 (corresponding to a p-value of 0.05) and binarizing the resulting spatial map to extract its volume.

#### 2.1.5. Impact Velocity and Location Estimations

The data gathered in Table 1 were analyzed to estimate the impact location and velocity in a fashion consistent with medico-legal expertise. Additional data were made available by neurosurgeon and paramedic.

- **Case 1:** An unwitnessed fall from a 2-story house roof, the victim sustained abrasions to the left forehead and face, a fracture to the left zygoma and a left intratrochanteric hip fracture, suggesting that the left frontal region made contact with the ground; the hip fracture suggests that the head was unlikely to have contacted the ground first. That forehead and facial grazes were in evidence, suggests a concomitant or subsequent involvement of the maxilla and or temporal bones and thus, a more diffuse, focal loading. The general description of the grazes suggests some tangential motion of the head, relative to a primary contact between the lower limb and the ground. This potentially was a result of angular motion of the trunk and upper body, relative to the contact of the lower limb. The provision of greater detail of the grazes may have informed the directionality and mechanics of the relationship between the head and contact surface further. The site and limited severity of the zygomatic fracture suggests that the head impact was relatively low energy, certainly compared to the potential for injury posed by a fall height through the distance presented in this case and that the surface was flat and firm, rather than irregular. Whether the surface was unyielding is unknown, however, given the overall low level of injury, the surface was probably not hard, i.e., neither concrete nor tarmac. The first point of contact was logically likely to have absorbed/dissipated a significant proportion of the impact energy. If it had been the head, a greater degree of injury severity might have been expected. However, a glancing head contact and subsequent lateral upper leg impact cannot be excluded. The fall height can be assumed to have been in the region of 5.7 m (typical height to the gutter of a 2-story house), the height of the gutter approximately 0.1 m, the assumed standing height of the accident victim, assumed 50*th* percentile male = 1.76 m, minus the 0.1 m distance from the top of the head to the likely point of contact around the “hat brim region.” Therefore, a total minimum fall height of 7.3 m is assumed, since by default, the assumption is that the male was standing at the lower edge of the roof, with no initial velocity and simply pitched forwards. Therefore, a simple fall is assumed, with no initial velocity or arc of rotation considered, through 7.3 m from which a maximum impact velocity is calculated to be 12 m/s. Forces which exceed the fracture tolerance limit in the literature are in the region of 5.35 kN (1,200 lbf), assuming an adult head mass of 6.82 kg (15 lb) and an acceleration of 80 g (Pappachan and Alexander, 2012). The fracture tolerance of the zygoma is in the order of 0.89–2.00 kN (200–450 lbf) (Pappachan and Alexander, 2012). Thus, the minimum velocity to produce fracture would be in the order of 2.24–5.02 m/s (5–11.25 mph). **The left frontal region is suspected to have made first contact with ground between 2.24 and 12 m/s**.
- **Case 2:** : Involved a typical “sucker punch” (or “king hit”) assault case (Patton and McIntosh, 2017). The male victim was punched to the face, which resulted in him falling backwards and striking his occiput on a rigid surface. The resulting head injuries may have been due to the punch, the fall, or a combination of both. A spectrum of punch response outcomes is possible, for example, if the punch had been delivered, such that little momentum (push) was transferred, producing a sudden loss of consciousness and no reflexive actions, then victim could have simply collapsed downwards and backwards, or downwards into sitting position and backwards. Alternatively, if the victim had been struck squarely, then momentum transfer would have produced an angular (arcing) motion of the upper body relative to his fixed feet, acting as a fulcrum. This would have resulted either in a relatively pure angular velocity about the fixed feet, if the legs had stiffened as a result of the blow, or alternatively, produced a combined linear and angular velocity if the legs had given way. Thus, a higher velocity and impact energy would have been produced. The worst case would be for a punch with significant transference of momentum such that the victim's straight body is submitted to translational velocity of 6.75 ± 0.27 m/s. The best case would be for the victim's body being slightly bent at waist with a translational velocity of 4.85 ± 1.33 m/s (Patton and McIntosh, 2017) **The victim is estimated to have hit his occiput on the ground with a velocity between 4.85 and 6.75 m/s**.
- **Case 3:** The victim was reported to have been kicked by a horse both to the abdomen and head and to have fallen backwards, prior to striking her head against the ground. Thus, the areas of impact were to the front, as a result of contact with the kick to the abdomen and head and back of the head (occipital region), due to the fall backwards. This was accompanied by a loss of consciousness for a brief period. Although the sequence was not specified, one could assume that the abdominal kick was first, since a kick to the head would have likely caused the victim to have initially fallen backwards away from any subsequent kick. With respect to the occipital impact, the velocity range is reported as being between a straight body translational velocity of 4.80 ± 0.22 m/s and a slightly bent at waist translational velocity of 3.78 ± 0.53 m/s (Patton and McIntosh, 2017). **In light of all the unknowns, an occipital impact velocity is estimated to have been between 3.2 and 4.8 m/s**.
- **Case 4:** The victim was a pedestrian hit by a van traveling at an estimated 20–30 mph. The head was reported to have contacted with the wing mirror before the victim was knocked to the ground. Frontal and occipital scalp degloving and significant arm and soft tissue injuries were produced. The primary impact velocity of the van cannot be directly attributed to the subsequent occipital contact with the ground, which can be assumed to be a result of a secondary impact, from momentum transfer producing a kinematic pedestrian response. If one were to consider just the vertical velocity of falling, one might consider the impact scenario similar to a crouched fall from standing, i.e., a slightly bent at waist translational velocity of 4.85 ± 1.33 m/s (Patton and McIntosh, 2017), though the degloving does suggest a more complex tangential component. If the head impact had been with a non-yielding part of the van, the fact that serious extra- and intracranial injury is absent would indicate an impact velocity to the front or rear of the head below 20 km/h (12 mph) (AL-Graitti et al., 2017). This is probably not the case here, since significant arm and soft tissue injuries were also reported. **Considering all these results in combination and comparing with other reported cases, the occipital region was probably impacted at velocity of between 4.17 and 9.72 m/s (15 and 35 km/h) (AL-Graitti et al.**, **2017****)**.
- **Case 5:** The victim was a pedestrian hit by a cyclist. Subsequent to a brief loss of consciousness, the victim had no recollection of the events prior to the accident. The right side of the head behind the ear impacted with the ground. A right posterior fossa epidural hematoma accumulated between the skull and dura, a consequence of skull fracture tearing an underlying blood vessel. This is frequently caused by a lateral force over the mastoid. Threshold velocity for impact related fracture data is of the order of 5 m/s (Gurdjian and Lissner, 1947; McIntosh et al., 1996; Yoganandan and Pintar, 2004). **As such, the impact is assumed to be on the right side of the head behind the ear at a velocity of 5 m/s**.
- **Case 6:** The victim was a pedestrian hit by a car traveling at approximately 30 mph. The right orbital/frontal region was impacted and fractured. This was followed by a loss of consciousness at the scene for at least 5 min. Fracture tolerance data does not exist for the facial fractures in evidence in this case. The mechanism of fracture is often associated with a “blow out,” a sudden increase in pressure in the orbit of the eye. This is attributed to an impact or impactor, which is larger than the orbital rim. The bones of the orbit are very fragile and no reliable fracture tolerance data exists. Whilst the fracture tolerance of the orbital rim is unknown, the frontal bone is the strongest bone of the face/head and since no fracture has occurred in this area, this suggests that a sub fracture level of loading has occurred. Force to the face is associated in the literature, assuming a head mass of 6.82 kg (15 lb) and an acceleration of 80 g [easily obtainable in a 13.4 m/s (30 mph) impact], with 5.35 kN (1,200 lbf), which exceeds the fracture limit of most of the facial bones (Pappachan and Alexander, 2012). The fracture tolerance of the frontal bones is in the order of 3.57–7.13 kN (800–1,600 lbf) corresponding to minimum impact velocities in the order of 8.9–17.88 m/s (20–40 mph) (Pappachan and Alexander, 2012). Thus, an absence of frontal bone fracture suggests impact velocities below this level. Since significantly lower average strength has been found for the female bone structure during impact experiments, the lowest values are considered. **As a consequence, an impact on the right orbital/frontal region at a velocity of 9 m/s is assumed**.
- **Case 7** The victim fell from a 3-story roof. The left temporal and frontal regions are reported to have made contact with the ground and a right wrist fracture, cervical and thoracic transverse spinous process fractures were also observed. The hand fracture, multiple rib and transverse process fractures suggest an impact to the back or side of the torso (no details provided about location), and a sacrifice related injury to the hand. As a result of a lack of detail, one can assume a superficial contact to the front/side of the head. That these injuries are as superficial as they are, given that cervical and thoracic transverse process fractures are in evidence, suggests that either the head made contact with a pliant surface, such as sand or soil, or that the significant impact energy was dissipated during an impact with the right hand and subsequently the side or back. The fall height can be assumed to be approximately 8.3 m (height to the gutter of a 3-story house), plus the height of the gutter approximately 0.1 m, plus the assumed standing height of the accident victim, assumed 50*th* percentile male = 1.76 m, minus the 0.1 m distance from the top of the head to the likely point of contact around the “hat brim region.” Therefore, a minimum fall height of 10.1 m is assumed, since again by default, the assumption is that the male was standing at the lower edge of the roof. Therefore, a simple fall with no initial velocity is assumed and no arc or rotation considered, with a height of 10.1 m producing an impact velocity of 14.1 m/s. **This analysis demonstrates that an impact to the left temporal and frontal region at a peak velocity of 14.1 m/s could have occurred, however, a lower velocity could be expected**.
- **Case 8:** The victim was charged by a bull running out of a cattle truck and thrown backwards against a metal fence and onto concrete, impacting his head, (occiput mainly), rendering him unconscious for a few minutes. Reverse engineering of a bull's velocity from the account provided, would require an appreciation of the bull's acceleration and velocity at the point of contact, which is not possible here. However, it is reasonable to assume that the victim's secondary impact velocity had to be at least as great as a simple fall backwards from standing, i.e., a straight body translational from standing velocity of 6.75 ± 0.27 m/s (Patton and McIntosh, 2017). Since there are facial fractures in evidence, and that the fracture tolerance of the frontal bones is 3.57–7.13 kN (800–1,600 lbf), corresponding minimum impact velocities are of the order of 8.94–17.88 m/s (20–40 mph) (Pappachan and Alexander, 2012). **As a consequence, an impact velocity at the occiput between 6.75 and 8.94 m/s is assumed**.
- **Case 9:** The victim was a cyclist knocked off a bicycle and reported to have landed on his head, such that his left parietal region made contact with the ground. This was accompanied by a left shoulder injury and multiple skin abrasions. Bitemporal contusions, traumatic subarachnoid hemorrhage and frontal and left parietal fractures were reported. A similar case was simulated and reported in the literature (Fahlstedt et al., 2012) with a resultant linear velocity of 5.3 m/s and a vertical velocity between 4 and 5.4 m/s. **As a consequence, an impact velocity of between 4 and 5.4 m/s on the left parietal region is assumed here**.

While some of these estimations are probably relatively accurate, an important proportion of these are extremely difficult to evaluate, due to an insufficiency of detail, and may be subject to very large variations. Those (e.g., case 7) were left anyway for the sake of discussion.

### 2.2. Mechanistic Simulations

The mechanistic simulations consisted of a FEHM submitted to different loading scenarios defined by predefined sets of impact boundary conditions. For each simulation, a mechanistic criterion was defined by the maximum shear energy rate each gray matter element of the head model experiences throughout the duration of the impact. This simulation was repeated with a range of loading scenarios to provide a library of pre-calculated damages.

#### 2.2.1. Finite Element Head Model

The FEHM is adapted from a previous version proposed earlier by Garcia-Gonzalez et al. (2018b), where it was validated for cranial impacts. It accounts for the gray matter, white matter with axonal anisotropy captured from diffusion tensor imaging (DTI), cerebrospinal fluid (CSF), skull, falx, scalp, and ventricles and consists of 2,354,594 tetrahedral elements. For each simulation, the boundary conditions were specified as described below, and a dynamic explicit simulation was run for 8 ms on Abaqus (ABAQUS Inc.). The mechanical behavior of each skull and gray matter element was tracked throughout each simulation. The von Mises stress was calculated in the skull elements and the shear energy rate was calculated in the gray matter elements. The maximum value experienced by each element throughout each simulation was recorded. A total of 445 simulations was run, of which 407 did not result in a fractured skull (see section 2.2.4).

One important assumption of this model is that only the inertia of the head contributes to the impact, and the rest of the body is left unmodeled. While this assumption has implications on the evaluation of the brain damage, the additional modeling of the body would be hampered by a lack of paramedical information. As such, this assumption is kept as a first approximation. Moreover , it is worth emphasizing that, under this assumption, the FEHM was validated against experimental data by means of acceleration-time curves for three impact conditions representative of real accidents and falls (see Garcia-Gonzalez et al., 2017 for more details): fall of a person from a bed; bike accident reconstruction; and experimental impact of human heads from cadavers against a rigid plate.

#### 2.2.2. Material Models

The constitutive framework originally developed by Garcia-Gonzalez et al. (2018a), and further extended for blast TBI simulations by Garcia-Gonzalez et al. (2018b), is taken as a basis. In this regard, the mechanical response of each tissue is decomposed into volumetric and shear components, leading to the definition of the Cauchy stress tensor as:

(1) $$\begin{array}{l} \sigma =\sigma_{vol}+\sigma_{iso} \end{array}$$

where **σ**_*vol*_ and **σ**_*iso*_ are the volumetric and isochoric Cauchy stress tensor components, respectively. This decomposition is also adopted to describe the total deformation gradient ***F*** as:

(2) $$\begin{array}{l} F=J^{1/3}F^{*} \end{array}$$

where *J* = *det*(***F***) is the Jacobian and F^*^ is the distortional part of the deformation gradient.

In this work, the skull, falx, CSF, and ventricles are modeled as proposed by Garcia-Gonzalez et al. (2017) for similar impact conditions: skull and falx as elasto-plastic materials with their corresponding material properties at the mean strain rate observed in the impact conditions tested (≈ 1 *s*^−1^); CSF and ventricles by the Mie-Grüneisen equation of state and a dynamic viscosity. Regarding the scalp, white and gray matter, these tissues are modeled by Garcia-Gonzalez et al. (2018b) by more sophisticated approaches based on hyperelastic theories to accurately describe nonlinearities. While the scalp tissue is defined in the exact same manner by a neo-Hookean model, the constitutive law for white and gray matter is modified to provide a more efficient solution for the specific impact simulations conducted here (the aforementioned work dealt with blast scenarios rather than head impact). The modified formulation for the total Cauchy stress contribution reads as:

(3) $$\begin{array}{l} \sigma =\frac{\mu_{m}}{J}\frac{1}{1-\frac{I_{1}^{*}-3}{j_{m}}}\text{dev}\left(B^{*}\right)\\ \;+\frac{2k_{1}}{J}\left(I_{4}^{*}-1\right)\text{exp}\left[k_{2}{\left(I_{4}^{*}-1\right)}^{2}\right]\text{dev}\left(F^{*}A_{o}F^{*T}\right)\\ \;-\frac{K_{o}}{\Lambda_{o}+1}\left[{\left(\frac{\rho }{\rho_{o}}\right)}^{\Lambda_{o}+1}-1\right]I \end{array}$$

where B^*^ = F^*^F^*T^, $I_{1}^{*}=\text{tr}\left(F^{*T}F^{*}\right)$, $I_{4}^{*}=\text{tr}\left(A_{o}F^{*T}F^{*}\right)$, *A*_*o*_ is the structural tensor providing axonal orientation within the white matter (see Garcia-Gonzalez et al., 2018b for more details), ρ is the current density and *I* is the second order unit tensor. Moreover, μ_*m*_, *j*_*m*_, *k*_1_, *k*_2_, *K*_*o*_, Λ_*o*_, and ρ_*o*_ are material parameters, whose values are provided for white and gray matter in Tables 2, 3. The calibration of the isochoric response of both white and gray matter was consistently performed accounting for the mean strain rate observed in the simulations. Note that this formulation, for the strain rate conditions observed in the simulations, is equivalent to the full original formulation published by Garcia-Gonzalez et al. (2018b).

**Table 2:** Constitutive parameters for white matter used in the simulations.

**Volumetric response**			
**ρ_*o*_ (*kg*/*m*^3^)**	***K*_*o*_ (*GPa*)**	**Λ_*o*_ (−)**	
**1, 140**	**2.19**	**6.15**	
**Isochoric response**			
μ_*m*_ (*kPa*)	*j*_*m*_ (−)	*k*_1_ (*kPa*)	*k*_2_ (*kPa*)
550	1.1	2.14	0

**Table 3:** Constitutive parameters for gray matter used in the simulations.

**Volumetric response**			
**ρ_*o*_ (*kg*/*m*^3^)**	***K*_*o*_ (*GPa*)**	**Λ_*o*_ (−)**	
**1, 140**	**2.19**	**6.15**	
**Isochoric response**			
μ_*m*_ (*kPa*)	*j*_*m*_ (−)	*k*_1_ (*kPa*)	*k*_2_ (*kPa*)
450	1.4	−	−

#### 2.2.3. Impact Boundary Conditions

**Figure 2:**
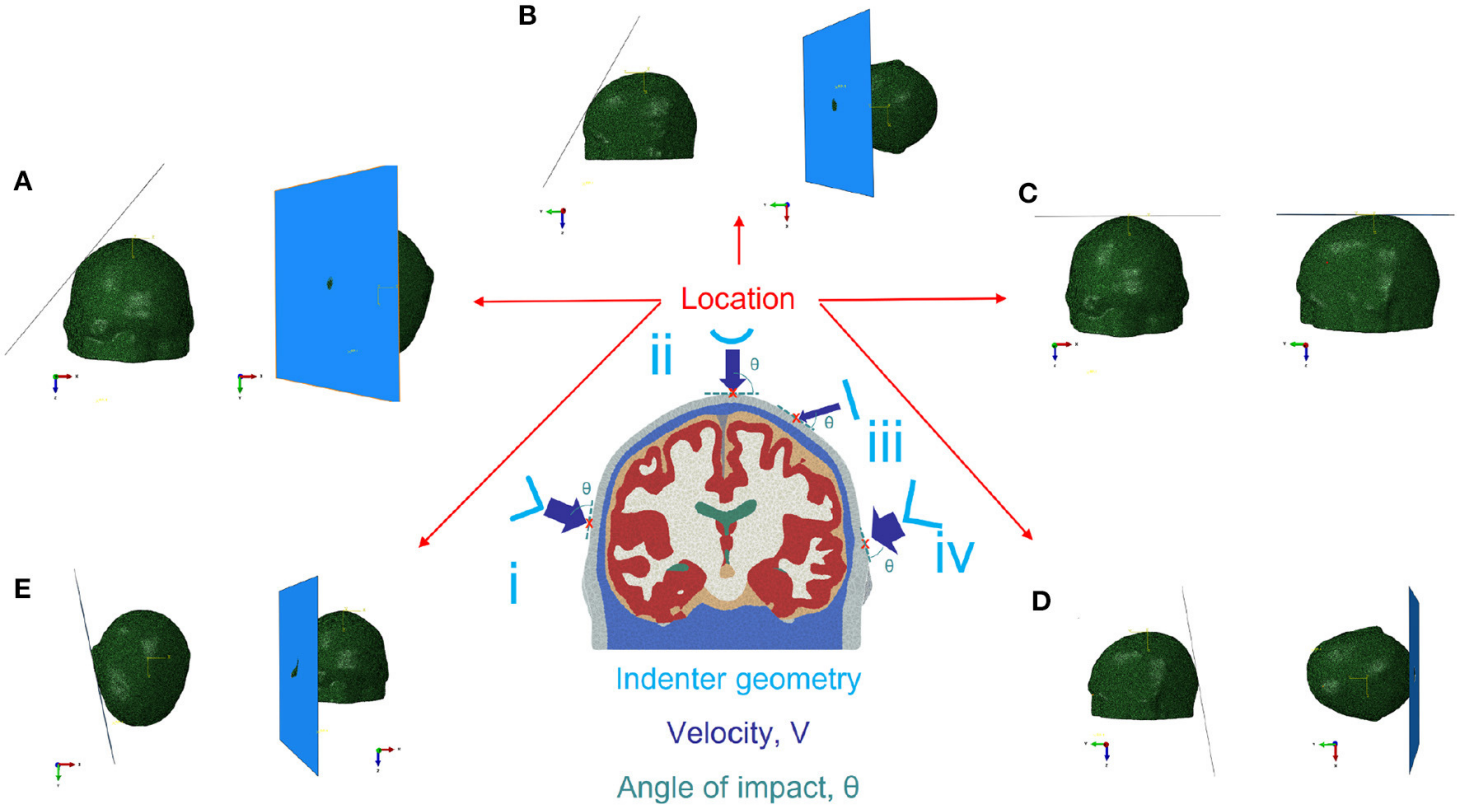
Impact boundary conditions for the FEHM; location: **(A)** lateral fronto-parietal, **(B)** fronto-polar, **(C)** vertex, **(D)** occipital, **(E)** temporal (the shown impactor corresponds to flat impactor iii); impactors: (i) blunt corner, (ii) round, (iii) flat, (iv) sharp corner.

Simulations were defined by a set of inputs for velocity, location of incidence, angle of incidence, and impactor geometry. The range of these boundary conditions were chosen so as to encompass the vast majority of impact cases, while avoiding velocities either *a priori* too small or too high to avoid extreme cases, e.g., no trauma or death on impact (see Table 4). All impactors were modeled as rigid, with a friction coefficient of 0.4 (Garcia-Gonzalez et al., 2017). For the “round” impactor, a cylindrical shape with a radius of curvature of 3.6 cm was used, while the “blunt corner” impactor was made of a right angle analytical surface smoothed along the edge with a 1 cm radius of curvature quarter of a cylinder, and the “sharp corner” impactor was made of an right angle smoothed with an edge of 0.3 cm. This determined the range of inputs for the library of pre-calculated FEHM simulations (see Figure 2).

**Table 4:** Impact boundary conditions.

**Boundary condition feature**	**Range**
Velocity	1 m/s < V <16 m/s
Locations of impact	(a) lateral fronto-parietal
	(b) fronto-polar
	(c) vertex
	(d) occipital
	(e) temporal
Angle of impact (from perpendicular)	−45° < θ <45 °
Indenter geometry	(i) blunt corner
	(ii) round
	(iii) flat
	(iv) sharp corner

#### 2.2.4. Mechanical Damage

All cases which resulted in a fractured skull were removed from the database. The skull was assumed to be fractured if more than 4% of all skull elements in the head model (4% corresponds to the percentage of skull elements spanning the maximum thickness of the skull) underwent a maximum von Mises stress exceeding the ultimate strength of bone (92.72 MPa; Wood, 1971).

**Figure 3:**
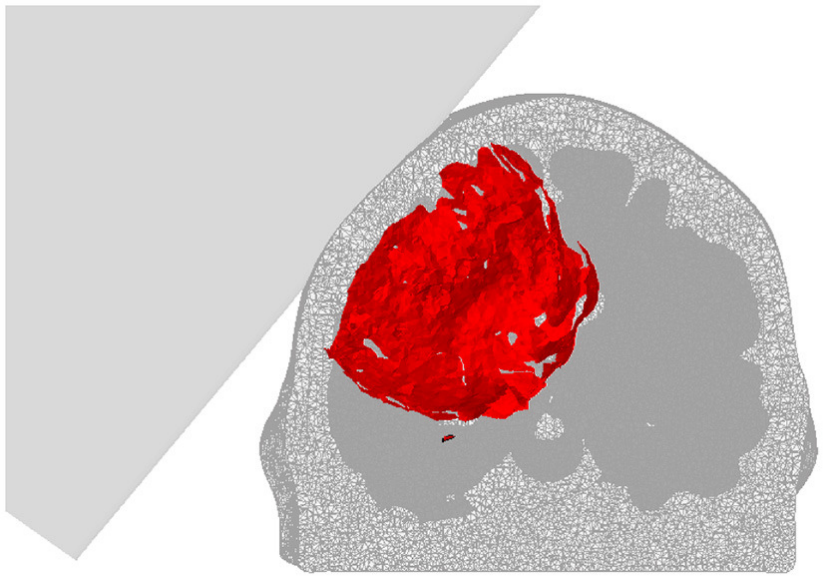
Isosurface of the damaged region (1*MJ*/*m*^3^*s* shear energy rate threshold) of the brain for a blunt corner impact, perpendicular velocity of 8 m/s, lateral fronto-parietal location at 1.6 ms after initial contact. Note that, while only the gray matter results are compared to the rsfMRI results, both white and gray matter are shown here.

A binary mask of the DMN mask was created by thresholding the template DMN mask from Smith and Nichols (2009) at a z-score of 3.1 (corresponding to a *p*-value of 0.05) and binarizing the resulting spatial map. The coordinate system of the finite element mesh was aligned with that of the fMRI images. The binary mask of the DMN (see section 2.1.4) was applied and all mesh nodes with coordinates within the DMN mask were extracted. Finally, all elements connected to these nodes were extracted to provide a DMN element set, and hence a mapping from the MRI domain to the element mesh. Damage to each gray matter element was determined by a material damage criterion. Previous studies of blast induced TBI suggested that a shear energy rate damage criterion of 100*MJ*/*m*^3^*s* in the gray matter provides a good correspondence to regions with oxidative stress in rat brains (Garcia-Gonzalez et al., 2018b). As both loading conditions and damage pathways are different (blast injuries and impact injuries have very different injury signatures), other thresholds were evaluated to match the functional criterion (see section 3). A final value of ≈ 1*MJ*/*m*^3^*s* was eventually chosen maximizing the correlation between mechanical and functional criteria. Figure 3 shows the isosurface of the damaged region of the brain for a blunt corner impact, perpendicular velocity of 8 m/s, lateral fronto-parietal location at 1.6 ms after initial contact (note that this case resulted in a damage of 50.22% to the DMN).

For each simulation in which the skull was not fractured, when the shear energy rate exceeded this criterion during the simulation, the element was assumed to be damaged. The percentage of damaged elements in the DMN was then calculated. This provided a library of pre-calculated loading scenarios on which the ML model could be trained and evaluated.

### 2.3. Machine Learning Layer

**Figure 4:**
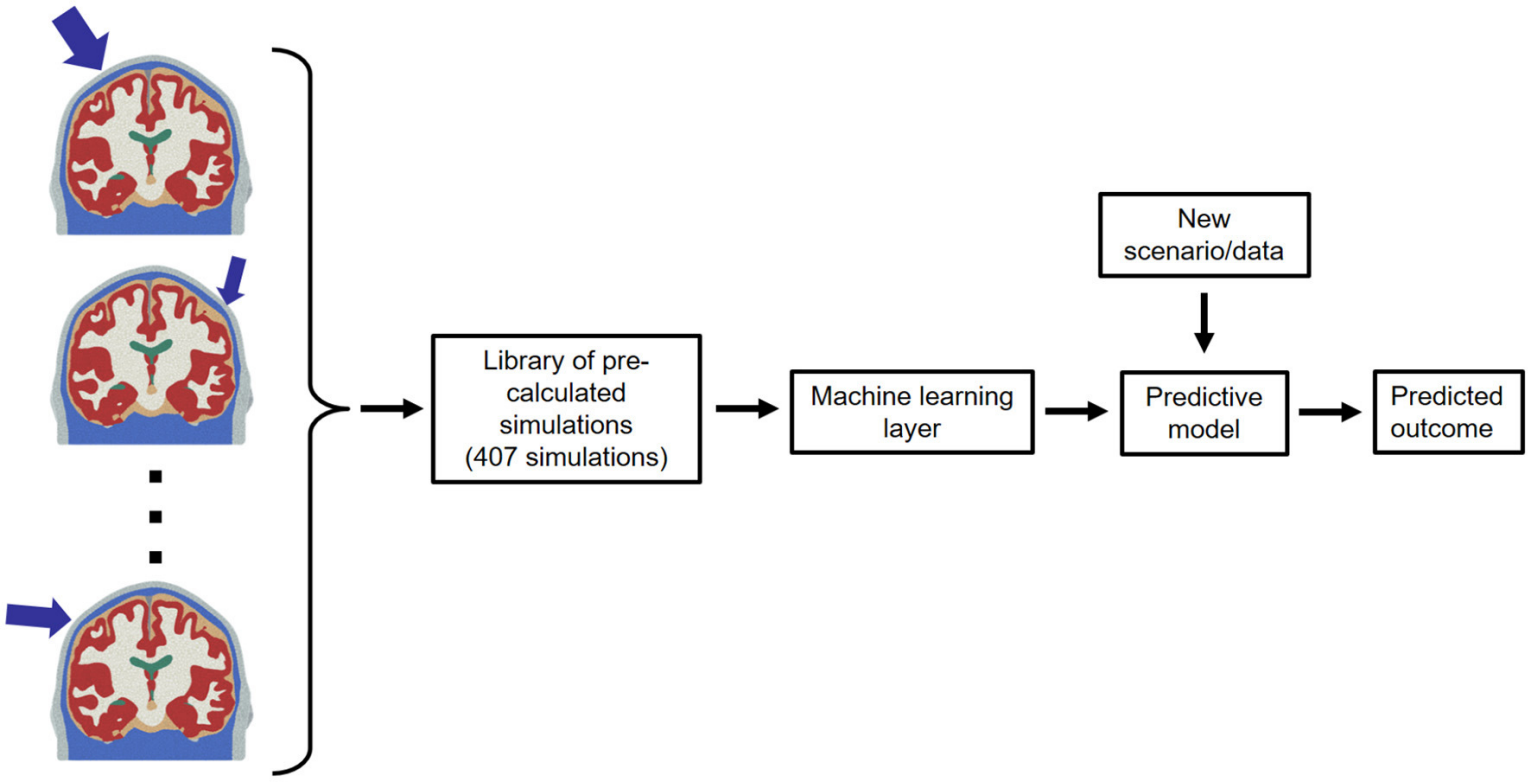
Schematic of the ML layer.

A ML layer was created to avoid the need to reproduce the FEHM simulations for each single scenario. To this end, the model was trained with 407 FEHM simulations for a range of combinations listed in Table 4. The overall approach and validation is explained below (see Figure 4).

#### 2.3.1. Machine Learning Algorithm

A ML layer was trained on binary outcome data to predict the probability that the extent of network damage exceeds a given threshold during an impact. In order to do this, a separate model was trained for each proposed threshold of network damage. The inputs used in the layer correspond to the inputs defined in section 2.2.3. Note that the shape of the impactor was represented by the radius of curvature of the impactor (the flat one was given a radius of 1 m). From these inputs and the FEHM, two additional features were extracted to be included in the ML layer inputs: distance from the point of impact to the closest element of the DMN; and angle between trajectory to the closest DMN node and trajectory of impact. To define the binary outcome, the DMN was considered damaged if the percentage of damaged gray matter elements exceeded the given network damage threshold.

The predictive ability of two ML approaches were compared in this paper. Logistic regression (Pregibon et al., 1981) was compared to a bagging ensemble method (Breiman, 1996). Although several other algorithms have been used to analyze TBI-related data (Siddiqui et al., 2015; Mitra et al., 2016; Minaee et al., 2019), logistic regression has previously been shown to outperform more complex models in TBI clinical outcome prediction (Steyerberg et al., 2008). The bagging method involves training a model with each of the following ML techniques: logistic regression (Pregibon et al., 1981), gaussian discriminant analysis (Fisher, 1936), k-nearest neighbor (Cover and Hart, 1967), Naïve Bayes classifier (Hand and Yu, 2001), and support vector machines (Boser et al., 1992). Given a test point, the ensemble method calculates the mean probability of damage from each of these trained models. This approach reduces the risk of incorrect classification and has been shown to outperform single algorithms (Dietterich, 2000).

A greedy forward feature selection approach was used to select statistically relevant input variables for each model; all other variables were excluded from the model. This was implemented with 5-fold cross validation and a fast algorithm (logistic regression) to reduce computational costs (Zhang, 2009). Feature selection was performed for each model independently. This resulted in a range of input variable sets dependent on the network damage threshold considered in each model.

In order to validate the ML pipeline and ensure robustness, the network damage threshold was set to a range of values (10, 30, 50, 70, 90%), and the model performance was assessed for each threshold. Performance was evaluated by leave-one-out validation (Wong, 2015). The area under the curve (AUC), sensitivity, and specificity were calculated for each model. When validating the model against the dummy and clinical datasets, the network damage threshold was set to the FEHM estimated network damage, and the clinically estimated network damage, respectively. This allowed the ML layer to predict the probability that at least the given proportion of the network was damaged.

Given the trained model and model inputs from nine clinical cases, the probability that mechanical damage exceeded the FEHM network damage estimation was predicted. In seven of the nine cases, there was a degree of uncertainty in the accident reconstruction, resulting in a range of mechanical damage predictions.

Finally, the ML models were used to predict the velocity at which the proportion of network damage is reached. For this analysis, the input velocity of each scenario was varied between 1 and 15 m/s whilst all other inputs remained constant. The predicted probability of reaching the network damage proportion was calculated for each velocity. This was plotted on a graph of probability against velocity. Because the ML model predicts only the probability that *at least* a given proportion of the DMN is damaged, the velocity at which the DMN is damaged by *exactly* this proportion can be understood as the velocity at which this plateau is *first* reached. This is assumed to be at ≈95% of the final plateau region.

#### 2.3.2. Dummy Validation

A series of experiments were carried out to simulate real-world accident scenarios to illustrate the comparison between direct FEHM and ML predictions of mechanical damage. These experiments provide a range of realistic inputs to the models.

The experiments involved a dummy falling down a set of stairs in a range of motions: forwards and backwards. Each fall was captured with video and motion capture software in Audiomotion Studios (Oxford, UK). The motion capture enabled accurate measurement of the velocity of impact, whilst video footage provided the location and angle of head impact and the impactor geometry. The dummy used in these experiments weighed 65 kg and was approximately 1.7 m tall, whilst the full height of the staircase was 2.07 m.

From these experiments, two scenarios were extracted, one fall forwards and one backwards, see Supplementary Videos 1, 2, respectively. In each of these scenarios, the stairs were the first point of contact for the head. In the forward fall, the head of the dummy impacted the (blunt) corner of the stairs in the fronto-polar region with a perpendicular impact velocity of 7.12 m/s. In the backwards fall the occipital region impacted the corner of the stairs with a perpendicular velocity of 7.69 m/s.

The FEHM, which simulated the impact, and the ML model were both used to predict damage to the DMN. These damage estimates were carried out independently from one another and the FEHM results were not used to train the ML layer. These damage estimates provided a means of comparing the FEHM outputs to those of the ML layer in a scenario used by the police and medico-legal community.

## 3. Results

### 3.1. DMN Functional Damage

**Figure 5:**
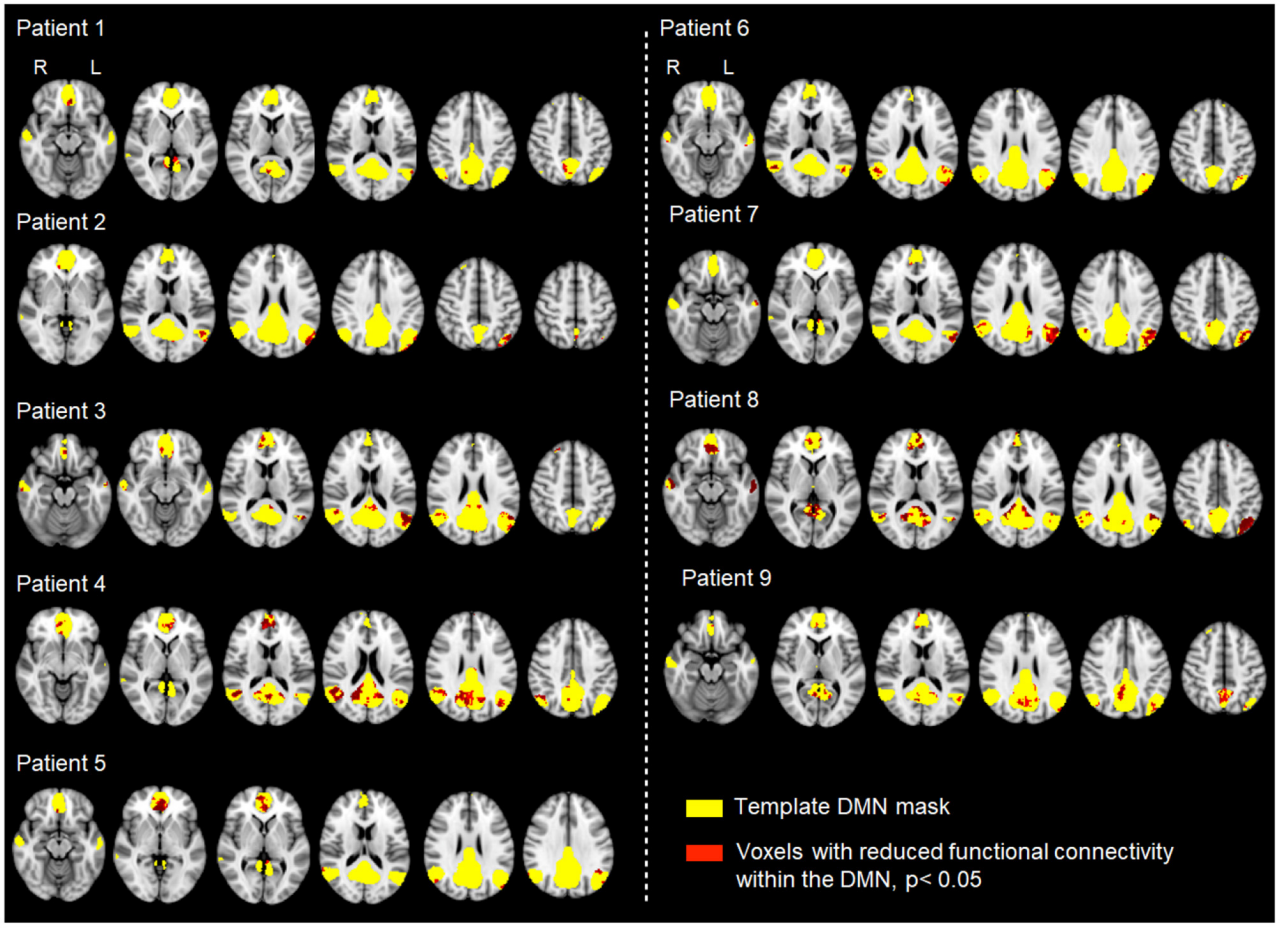
Analysis of functional connectivity (rsfMRI signal correlation) within the DMN across nine patients with varying degrees of TBI and different mechanisms of injury. Individual patient resting data were compared to 18 controls using permutation testing. Each map shows the patient-specific threshold-free cluster-enhancement t-statistic images, depicting all voxels with significantly lower functional connectivity (*p* < 0.05) than the corresponding values in healthy controls.

The DMN resting brain network was successfully identified from MRI scans conducted in the hyper-acute phase through dual regression in each of nine TBI patients and all 18 healthy controls. The “disconnectivity” within the DMN, i.e., the proposed functional damage parameter, varied substantially across the nine patients (see Figure 5), ranging from 1.5 to 19.4% (see Table 5).

**Table 5:** Proportion of functional damage in the DMN as evaluated from functional correlation disruption.

**Cases**	**1**	**2**	**3**	**4**	**5**	**6**	**7**	**8**	**9**
Damaged DMN (%)	1.48	3.2	5.17	14.67	4	2.53	8.93	19.43	6.17

### 3.2. Numerical Model Performance

#### 3.2.1. Machine Learning Layer Performance

Tables 6, 7 provide a comparison of model performance when implementing a bagging ensemble method and logistic regression, respectively. These models were validated over a range of network damaged proportion thresholds. All models provide good discrimination, with AUC values consistently >0.975 across all damage thresholds. The bagging method outperformed the use of logistic regression alone. The average AUC across all network damaged proportion thresholds was 0.985 for the bagging method, and 0.981 for logistic regression.

**Table 6:** Bagging method performance for a range of network damaged proportion thresholds.

	**DMN damaged proportion threshold (%)**				
	**10**	**30**	**50**	**70**	**90**
AUC	0.987	0.986	0.986	0.989	0.976
Brier's score	0.052	0.046	0.034	0.027	0.030
Sensitivity	0.752	0.702	0.790	0.829	0.731
Specificity	0.983	0.988	0.988	0.981	0.976
Accuracy	0.921	0.958	0.958	0.966	0.961
Dataset balance	0.268	0.206	0.1523	0.101	0.064

**Table 7:** Logistic regression method performance for a range of network damaged proportion thresholds.

	**DMN damaged proportion threshold (%)**				
	**10**	**30**	**50**	**70**	**90**
AUC	0.979	0.981	0.978	0.988	0.979
Brier's score	0.056	0.048	0.034	0.027	0.028
Sensitivity	0.817	0.762	0.823	0.829	0.615
Specificity	0.956	0.966	0.986	0.986	0.987
Accuracy	0.919	0.924	0.961	0.971	0.963
Dataset balance	0.268	0.206	0.1523	0.101	0.064

Dataset balance identifies the proportion of simulations which resulted in damage to the brain's network. In most metrics, results were unbiased by the dataset balance, with AUC and Brier's scores remaining relatively constant. However, the dataset balance had an impact on the model's sensitivity, its ability to predict the cases that resulted in damage to the DMN. On average, the bagging method provided improved AUC to that of logistic regression, and was thus used subsequently.

#### 3.2.2. Dummy Validation

In the two dummy fall scenarios, damage to the DMN was predicted by both full FEHM simulations and the ML model. Table 8 shows the resulting damage and velocity predictions from these two approaches. The predictions of 64.1 and 24.9% are the proportions of elements in the DMN region having reached the threshold of shear energy rate of 1*MJ*/*m*^3^*s* in the direct FEHM simulations for the forward and backward impacts, respectively. The ML probabilities correspond to the predicted probability that the two impact scenarios would lead to, at least, those proportions, i.e., the ML layer predicts that there is 50.6 and 72.7% of chance that the impact damages at least 64.1 and 24.9% of the DMN region, for the forward and backward impacts, respectively.

**Table 8:** A comparison of FEHM and ML mechanical damage prediction for two dummy fall scenarios: “FEHM prediction” is the proportion of the DMN region damaged according to the finite element simulation, “ML probability” is the ML-predicted probability that “at least that much DMN region is damaged,” “ML velocity” is the ML-predicted velocity at which there is 95% chance that the FEHM-predicted damaged proportion is reached.

**Fall motion**	**FEHM**	**ML**	**ML**
**(impact velocity)**	**prediction (%)**	**probability (%)**	**velocity (m/s)**
Forwards (7.12 m/s)	64.1	50.6	≈8.6
Backwards (7.69 m/s)	24.9	72.7	≈7.9

**Figure 6:**
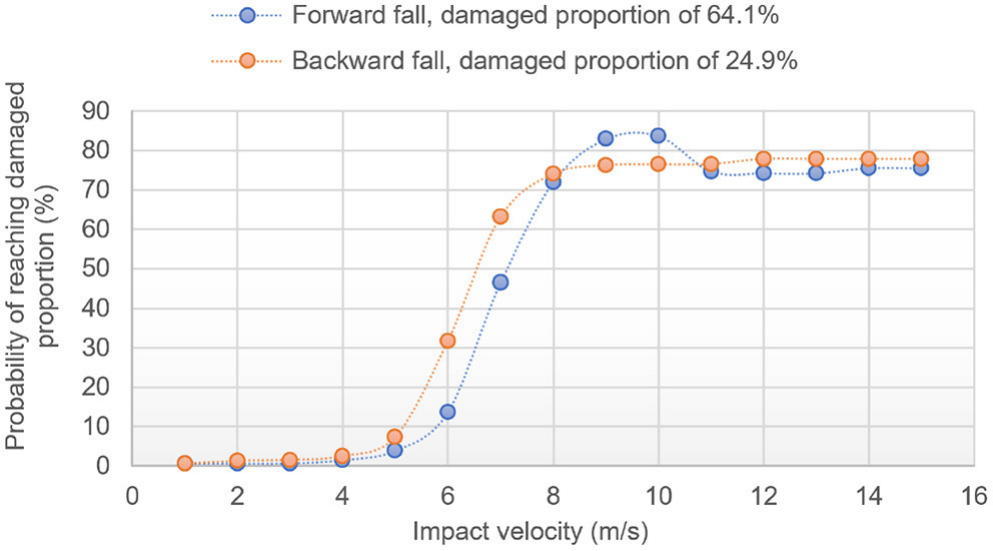
ML predicted probability of damaging at least 64.1% of the DMN region in forward fall and 24.9% in backward fall for different impact velocities.

Figure 6 offers another way to use the ML model by showing the probabilities that these proportions are reached for a range of potential impact velocities, for both scenarios. Both curves are sigmoids with plateau regions of ≈83% (≈75% if considering the last portion of the plateau) and ≈76.5%. Assuming that the plateau is first reached at ≈ 95% of the plateau value, the ML model predicts that reaching 64.1 and 24.9% a damaged proportion would occur at ≈8.6 m/s (≈8 m/s if considering the last portion of the plateau) and ≈7.9 m/s for the forward and backward falls, respectively (see Table 8). In this graph, the sigmoid never reaches 100% probability. This is due to nature of the ML methods, which are unlikely to estimate 100% probability that the given network damage threshold has been reached.

While an overall good match is confirmed between the FEHM and the ML model in this “real life” scenario, it is worth emphasizing that, because of the nature of the sigmoid shapes, velocity predictions for a given proportion of damaged DMN are less subject to noise error than the probability predictions for a given impact velocity. Another point is that the ML layer is bound to struggle at high velocities/high proportions because of the smallest population of training data having such large damage; this explains why the sigmoid curves might oscillate in the upper plateau region.

### 3.3. *In silico* Model Prediction

#### 3.3.1. Input Sensitivity

**Figure 7:**
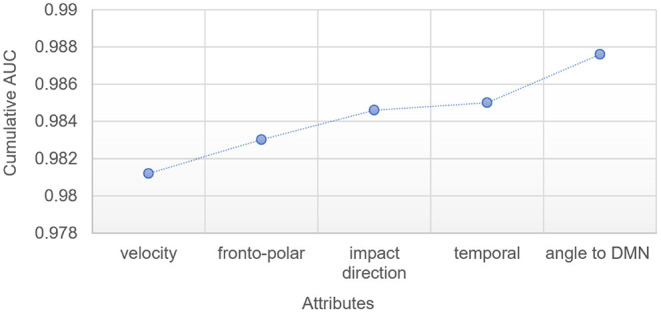
Improvement in ML model AUC with each additional attribute, for ML model trained at a 50% threshold.

A feature selection algorithm was implemented to identify the most predictive model inputs. Figure 7 highlights the improvement in model performance with each additional input when the model is trained at a 50% threshold. Velocity was selected as the most predictive attribute, providing an AUC of 0.985 when used alone to predict network damage. Whether the fronto-polar region was impacted, whether the impact was perpendicular to the head, and whether the temporal region was impacted, best improved the prediction in this order, with the angle between impact location to closest DMN node, and impact direction finally allowing the AUC to reach a value of ≈0.988.

#### 3.3.2. Clinical Validation

In this section, the conditions established in section 2.1.5 were used as inputs for the ML model. The same methodology described in section 3.2.2 was used, but instead of taking as input the DMN damaged proportion as predicted from FEHM simulations, the proportion of damaged DMN calculated from the proposed functional criterion (see section 2.1.4) was used instead. Table 9 shows the ML predicted probabilities that the clinically predicted damaged DMN proportion (see Table 5) was reached for the velocity ranges evaluated in section 2.1.5 for all nine patients.

**Table 9:** Proportion of functional damage in the DMN as evaluated from correlation disruption, ML predicted probabilities that at least this proportion is reached for the manually estimated range of impact velocities, and ML predicted impact velocities at which 95% of the final plateau probability *P*_*f*_ is reached for the clinical DMN damaged proportion, for the nine patients (see section 2.1.5 and Figure 2).

**Cases**	**Functionally**	**ML predicted probability (***P***)**	**ML predicted**
**(conditions)**	**damaged**	**range for impact velocity (***V***)**	**velocity**
	**DMN (%)**	**range evaluation**	**at 95% *P*_*f*_ (m/s)**
1	1.48	5.2% < *P* < 91.1%	
(b-iii)		(2.24*m/s* < *V* < 12*m/s*)	≈ 6
2	3.2	17.5% < *P* < 70.7%	
(d-iii)		(4.85*m/s* < *V* < 6.75*m/s*)	≈7.5
3	5.17	6.0% < *P* < 30.5%	
(d-iii)		(3.25*m/s* < *V* < 4.8*m/s*)	≈7.4
4	14.67	2.6% < *P* < 97.0%	
(d-iii)		(3.52*m/s* < *V* < 9.72*m/s*)	≈7.1
5	4	*P*≈36.0%	
(a-iii)		(*V*≈5*m/s*)	≈6.9
6	2.53	*P*≈95.3%	
(b-iii)		(*V*≈9*m/s*)	≈5.9
7	8.93	*P* < 76.5%	
(e-iii)		(*V* < 14.1*m/s*)	≈7
8	19.43	56.0% < *P* < 77.0%	
(d-iii)		(6.75*m/s* < *V* < 8.94*m/s*)	≈7.9
9	6.17	18.0% < *P* < 71.4%	
(a-iii)		(4*m/s* < *V* < 5.4*m/s*)	≈6.9

**Figure 8:**
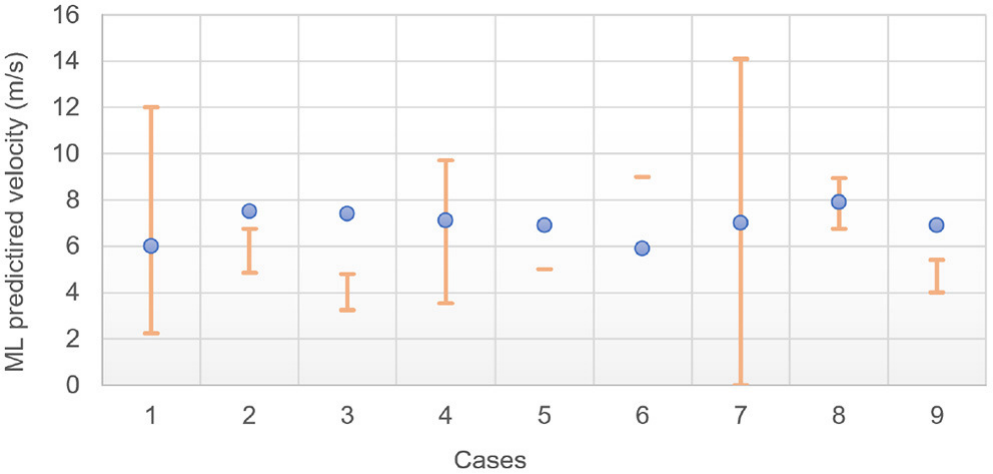
ML predicted impact velocities at which 95% of the final plateau probability *P*_*f*_ is reached for the clinical DMN damaged proportion; orange bars are the velocity range estimates from the analysis of section 2.1.5.

As highlighted in section 2.3.2, the impact velocity prediction for a given damaged DMN proportion is prone to greater error than the damaged DMN proportion prediction for a given impact velocity. In addition, the probability refers to the fact that *at least* a given proportion of DMN is damaged. As such, while case 1's results point toward a velocity of impact most likely to be toward the end of the range (12 m/s), it is not clear whether the probability of 91.1% for 12 m/s sits in the plateau region of the sigmoid, i.e., if a lower velocity would also reach such high proportion. To avoid this difficulty in the interpretation of the results, the sigmoid curves of the ML predicted probabilities of reaching the clinically evaluated damaged DMN against different impact velocities were plotted for all nine cases (not shown here). For each one of them, the velocity at which 95% of the plateau probability is reached was extracted. This value corresponds to the velocity at which the clinically evaluated damaged DMN proportion is first reached according to the ML model. The results are compared against the “manually” estimated range of velocity of section 2.1.5 in Figure 8 and Table 9.

## 4. Discussion

### 4.1. Model Limitations

#### 4.1.1. Head Model Dependence

The FEHM used here was originally developed from high resolution anatomical T1 and T2-weighted MRI images of a subject available from the Human Connectome Project (HCP Subject ID: 100307) (Essen et al., 2013; Garcia-Gonzalez et al., 2018b). Ideally, one would use a dedicated FEHM for each individual to offer a more tailored solution to the damage prediction by accounting for morphological differences between patients. Because of the time it would take to develop such models (on-the-fly in the context of clinical admission), and despite some recent advances in this direction (Li et al., 2020), such a solution remains impractical. Additionally, due to the very nature of the ML layer, which first requires training on a library of FEHM simulations, doing so would not allow for ML prediction. It is also worth mentioning that having a morphologically correct head model scanned before injury for any TBI patient is unrealistic. An alternative would be to create a finite library of population-wide representative head morphologies, which would constitute one of the inputs of the ML layer. This would however require much larger libraries for the training of the layer, if one were to account for sex, age, etc. A direct comparison between three different head models has shown significant disparities in the brain mechanical response in nearly all brain regions of the models (with the caveat that these head models were all idealized and not constructed from imaging) (Ji et al., 2014). More recently, the study of more realistic head models for different morphologies has reached similar conclusions (Li et al., 2020). However, in the former study, the models showed similar trends in the relationship between mechanical response and kinematic response, indicating that a given model can be used independently of the others for a given set of impact conditions as long as it is used consistently. While not excluding the possibility to include more flexibility in the morphological variations between patients in some future work by using, e.g., novel morphing approaches (Li et al., 2020), the approach consisting of using only one model thus seems justified as a first approximation, while allowing for faster ML predictions.

Each FEHM requires a set of constitutive models for the different regions identified within the head (typically, gray and white matter, skull, CSF as a minimum). Those need to be chosen carefully depending on the level of detail (e.g., homogenized brain vs. independent white and gray matter) but also loading conditions. For instance, blast loading conditions would typically require equations of state to adequately capture the volumetric response under shock waves and the viscous-relaxation processes can *a priori* be ignored for very short time-scales (Moore et al., 2009), while slow loading scenarios, such as in the second stage of labor, when the head of the foetus is being compressed in the vaginal canal, would ideally require viscoelastic laws to capture the fetal head molding of the infant head (Ami et al., 2019). Any intermediate situation, such as the ones considered here, would need to balance the need for time-dependent models against the timescales involved, along with other features more or less relevant depending on the leading deformation mechanisms: whether viscoelastic models are required, whether tissue damage and/or fracture should be accounted for, whether tissue anisotropy is relevant, etc. Here, a careful analysis of the most salient features was carried out, and each region of the used FEHM was assigned a constitutive model with parameters identified for the range of loading considered in this work. While more work is required to ensure that each chosen constitutive model and its associated material parameters are indeed optimum, the proposed setup is believed to constitute a first good approximation. It must finally be noted that as better models and material parameters are identified, the overall framework function remains the same and those new changes would be trivial to incorporate.

#### 4.1.2. Kinematics

In the approach followed here, the kinematic behavior immediately after impact is assumed to be solely driven by the inertia of the head, i.e., the contribution of the rest of the body, and in particular, the neck is not accounted for. This approach has been proven to be deficient in some cases (Wang et al., 2020). While it could be argued that it could still be considered as valid in cases where the inertia of the body does not contribute to the impact (e.g., if one falls sideways, and/or is hit directly at the head), or when the neck does not hamper the movement of the head (e.g., during and immediately after the impact of an unaware or unconscious individual), it remains an inaccurate representation of the real-life impact. An ideal simulation would couple a multibody dynamics simulation to the proposed FEHM to ensure that the kinematic behavior of the head is more accurately modeled. It must, however, be emphasized that the more complex the underlying mechanistic model is, the more inputs a given ML layer would have to incorporate. Therefore, while having a set of impact conditions on the head, as done here, can easily be incorporated in the ML layer, incorporating inputs related to the entire body based on clinical information from the scene is realistically currently unworkable.

#### 4.1.3. Skull Fracture

A final limitation of the FEHM is that, while the onset of skull fracture was predicted, its mechanical deformation post-fracture was not modeled. As such, the choice was made to train the ML layer exclusively on simulations which did not result in skull fracture. However, five out of the nine patients studied in this work experienced skull fracture (cases 1, 6, 7, 8, and 9), and, while those were not judged to be important enough to influence significantly the brain deformation in those cases (e.g., left zygoma fracture for case 1), it is clear that better predictions would be expected with additional fracture mechanistic features embedded in the FEHM for a more general applicability.

### 4.2. Predictive Accuracy

The ML layer has been shown to be very effective in the prediction of the simulation behavior (with AUC values all above 0.97 in the worst case), especially considering the reduced number of simulation scenarios. This prediction could be enough for some preliminary clinical assessment. An eventual high-fidelity ML prediction with additional inputs could be leading to some overfitting, owing to the relatively general nature of the mechanistic model. The proposed approach is a trade-off between the descriptive power of the simulation and the granularity of the ML predictions. According to this, the number of features and the feature selection procedure are tailored to the overall complexity of the ML tasks (in number of instances and features). As seen in section 3.3.1, a single feature already provides a reasonable high accuracy level. Additionally, the characteristics of the data also constrain the use of a given ML algorithm. More advanced techniques, such as neural networks (e.g., deep learning as an extreme case) are designed for two or more higher orders of magnitude in the number of simulations to analyze.

Another interesting aspect is the stability of the results independently of the DMN damage proportion threshold (see Tables 6, 7). Indeed, from 10% threshold up to 90% threshold, there is a ×4 factor in the ratio of the minority class (0.064–0.268 for 90 and 10%, respectively). In all cases, neither the AUC nor the sensitivity or the specificity are compromised.

The stability of the sensitivity and specificity is of particular importance in the clinical setting. Sensitivity would be crucial to enable identification of network damage within the DMN in the acute or hyper acute phase following injury. Specificity would allow clinicians to rule out the possibility of injury enabling decisions regarding discontinuation of neuro protective interventions. Tables 6, 7 show that the specificity consistently performs higher than the sensitivity for both models. Future ML models could be tuned to ensure that the specificity is not maximized at the expense of the sensitivity.

In the future, both the mechanistic simulations and the ML layer should become more detailed. This also means that the number of required simulations should become larger but also the number of descriptive features (now constrained to the primary characteristics: velocity, location, and angle). In addition, other derived indicators shall be obtained and other topological and spatial considerations shall be included.

### 4.3. Clinical Data

One of the main limitations of this work is the relative scarcity of clinical data. However, the data were acquired within the first 24 h of head trauma, including severe injuries. This quick-paced availability requires a specialist center, able to acquire data in patients who are ventilated and intubated. For logistical reasons such data are therefore exceptionally difficult to acquire in large volumes. While our sample size is limited for this reason, the type of data presented here is precisely what is required to make realistic predictions in a clinically meaningful (“hyper-acute”) time period. As such, balancing data quality and data quantity was a necessary challenge in this work. By providing here a novel framework with proactively gathered (albeit limited) data, the goal of this work is to emphasize the need for established widespread protocols for data curation in a proactive model driven fashion, as opposed to models making use of limited data available after their independent retrieval, usually from much later time-periods after injury, and likely brain recovery processes, have occurred.

It is finally worth emphasizing that our patients were followed for 6–9 months, which could offer further development to the model predictions in future work.

### 4.4. Resting State Network Relevance for TBI Prediction

Despite advances in the care of patients suffering TBI, long-term clinical and neuropsychological outcomes are often poor, irrespective of apparent injury severity (Brooks et al., 2013; Stocchetti and Zanier, 2016). MRI studies performed in the days, weeks, and months following TBI have uncovered a crucial role played by diffuse axonal injury (DAI) in the long term clinical, functional and neuropsychological outcome (Tong et al., 2004; Li and Feng, 2009; Skandsen et al., 2010). Midline structures, including the corpus callosum and cingulum bundle are particularly susceptible to the shearing forces causing DAI (Yount et al., 2002; Chan et al., 2003). Since high level cognitive functions such as memory, attention and executive function require the integration of information processing across spatially distinct brain regions, it has been proposed that DAI induces cognitive impairment by disconnecting distributed brain networks (Inglese et al., 2005; Niogi et al., 2008; Kinnunen et al., 2010; Bonnelle et al., 2011).

rsfMRI is not, *per se*, optimum to measure functional activity, given that one cannot be certain of what function is being measured (this applies especially in the context of the DMN, which “shuts off” during tasks). However, this remains to date the only method available for use in severely head injured patients, many of whom were intubated and ventilated during scanning. rsfMRI allows measurement of slow neuronal signal fluctuations without the need for a task, enabling the study of functionally relevant brain networks in TBI patients regardless of the severity of injury. In this way, the use of rsfMRI enabled us to measure functional brain networks in patients among our cohort who were intubated and ventilated during MRI scanning. Among brain networks known to be disrupted following TBI (Stevens et al., 2012), the DMN has received particular interest due to its proposed role in the development of attentional deficits (Raichle, 2010; Bonnelle et al., 2011; Sharp et al., 2011) which often follow DAI (Scheid et al., 2003; Povlishock and Katz, 2005). The brain regions that make up the DMN (Raichle et al., 2001) are particularly susceptible to DAI, including notably the midline posterior cingulate cortex, precuneus, and ventromedial prefrontal cortex alongside the inferior parietal lobe, lateral temporal cortex, and hippocampal formation. Crucially, the regions of the DMN show highly correlated brain activity at rest.

Previous studies report differing DMN functional connectivity according to severity of injury and timing of imaging (Zhou et al., 2012; van der Horn et al., 2017). Here, we show that DMN disruption can be identified within the first 24 h following trauma, using an objective statistical metric sensitive to network disruption at the single patient level. We propose that our damage load metric offers advantages over typical group-based studies in understanding and predicting the effects of trauma. Group-based or population average studies, by definition, aim to identify features that are common across patients. Such approaches consequently discard the fundamental heterogeneity in head injury mechanisms and their downstream network impact that likely account for vast differences in outcomes among individuals.

### 4.5. *In silico* TBI Prediction

#### 4.5.1. Coupling of Causality and Correlation

Figure 7 shows the five more important attributes in the ML layer per increased order of contribution to the prediction of the layer when used at a 50% threshold. Unsurprisingly, the velocity of impact is the most important factor. Whether or not the impact location is in the fronto-polar region or the temporal region are the second and fourth most important attributes, with the third being whether the impact was perpendicular to the head. Finally, the angle between impact location to closest DMN node and impact direction allows for a slight increase in the predictive ability.

From a geometrical perspective with respect to the DMN nodes, an impact location in the fronto-polar should indeed *a priori* have a stronger influence on the DMN than a temporal impact. The relative importance of the angle to DMN (by 0.3%) is slightly more surprising, especially considering the fact that the binary attribute indicating whether the impact is perpendicular or not was already selected as the third most important attribute. This particular trait demonstrates the advantage to couple mechanistic simulations with ML. In this case, the mechanistic FEHM simulations incorporate indirectly information related to the angle between impact direction with respect to the closest DMN node. A ML layer on its own would not be able to incorporate information of this kind without additional preprocessing of the head morphologies and mechanical features of stress wave propagation with respect to impact direction. Such complementarity of causality (through the mechanistic simulations) and correlation (through the ML layer) has already been advocated as an ideal way to incorporate physical mechanisms in a scalable fashion (Baker et al., 2018). This work demonstrates that additional information driven by a mechanistic understanding of the physical processes at play during tissue damage can indeed allow for additional predictive power in the ML layer.

It is worth noticing that, for each given damaged DMN proportion, a new training session is needed. This means that, while Figure 7 only shows the results for a 50% threshold, each new threshold, and thus each curve in Figure 6, will select a new set of attributes to work with. For two of the nine patients of this study, training did not use the angle to DMN but selected the fact that the impactor is or is not perpendicular (results not show here). In all cases however, the velocity of impact was the main attribute followed by either the shape of the impactor or the angle to DMN.

Overall, this dynamic feature selection offers an individualized prediction of the impact on brain function based on a given head injury. These predictions shed light onto the nature and extent of likely associated tissue disruption in an individual patient that is not captured by current clinical assessments. In this way, models able to predict down-stream functional outcomes from early paraclinical metrics offer potential to optimize treatment at a time when crucial clinical decisions need to be made.

#### 4.5.2. Predictive Performance

The method proposed here postulates a direct relationship between mechanical damage and functional damage. It can be used in two ways: (i) it can assess the probability that the DMN has been damaged to the extent measured clinically for a given impact, (ii) or it can predict a velocity at which such extent of damage can be reached, assuming one knows the remaining boundary condition attributes (impact location, angle, etc.). This approach was tested for nine patients whose impact conditions were estimated from paramedical and clinical notes in a manner consistent with medico-legal methodology.

The shear energy rate damage criterion was taken to be at ≈ 1*MJ*/*m*^3^*s* (see section 2.2.4). The quantitative evaluation of DMN damage proposed here is a novel approach whose correlation with mechanical damage has never been attempted. Garcia-Gonzalez et al. (2018b) successfully observed a correlation with oxidative stress in the context of blast injury for a much larger value of the shear energy rate damage criterion, but as loading conditions and damage pathways are different (blast injuries and impact injuries have very different injury signatures), another value needed to be estimated. The proposed threshold of ≈1*MJ*/*m*^3^*s* is interestingly close to the axonal deformation energy rate threshold of 1.5*MJ*/*m*^3^*s* for oxidative stress in blasted white matter (Garcia-Gonzalez et al., 2018b). While white matter damage was not predicted here for lack of experimental comparison (rsfMRI measures gray matter activity), indirect damage of white matter might also directly influence the rsfMRI results, and the proposed model could be benchmarked in future work against DTI data to assess damage in the white matter tracts. This could also be done indirectly by measuring the correlation (or lack thereof) of the DMN with the rest of the brain. It is finally important to note that the results obtained here intrinsically depend on this threshold calibration. However, to confirm with sufficient significance that the value chosen here is indeed the right one, a much larger dataset of patients would be needed. Future work shall focus on gathering such data.

The two predictions made by the ML method are assessed in Table 9 and Figure 8. Firstly, the model should be able to assess the probability that the DMN exceeds a given threshold. When the threshold of the model was set to the clinically observed network damage, an ideal model should provide a high probability that the network is damaged for the given scenario. In Table 9, seven out of nine cases produce a probability of damage over 70%, however some probabilities of damage range from small to large values, for example in cases 2 and 4. This reflects the difficulties faced in estimating the impact scenarios from parametric data, which often resulted in a large range of possible impact velocities.

The model also provides an estimate of the velocity at which the clinically observed network damage was met. As shown in Table 9 and Figure 8, four out of nine patients' ML predicted velocity is within the range manually estimated. As indicated earlier, a few of these cases did not have enough information to allow for a confident estimation; very rough values were still proposed in the interest of discussion. All patients presented significant TBI and the model predicts that the range of velocity expected to lead to such TBI is much narrower than manually evaluated. In particular, values of impact velocity between 6 and 8 m/s for all nine patients are expected, while the manual estimation of the range was six-fold larger. Note, however, that different ML training designs could be used to better estimate velocities. In particular, a backward estimator (from the damage to the characteristics of the impact) could be used instead of the forward model proposed here (from the features to the predicted damage).

#### 4.5.3. Forensic Relevance

Establishing whether a traumatic head injury is a result of an accidental or non-accidental cause is a fundamental question in forensic investigations. Often, practitioners are provided with only a brief third-party description of a causal event and struggle to establish a sufficiently detailed understanding of a cause and effect relationship with which to make a differentiation. Current medical understanding, acquired by training, anecdote, and experience is supplemented with scientific evidence, drawn from specialities such as pathology, radiology, and population-based epidemiology. The head and central nervous system may be injured by many different mechanisms; therefore, developing a necessary understanding of the cause from practical experience and epidemiology alone is a significant challenge, since there are very many biomechanical variables that require consideration.

A retrospective biomechanical engineering analysis can assist a forensic investigation by providing cause and effect understanding with regard to a stated or inferred injury-causing event. This can be undertaken by characterizing the biomechanical loading environment during the event in question, quantifying the physical loading conditions and evaluating their potential to produce injury by, where possible, drawing comparisons with injury tolerance and/or epidemiological data.

Given the wide range of velocities, locations, angles, and materials associated with head injury mechanics, it is unrealistic to anticipate that a single injury risk metric can exist for every possible scenario. Specific to the head, one primary reason is the very many different motions that can occur when a head is struck with an object, or when a head strikes a surface and/or is whiplashed, since the complex variety of potential responses makes each injury-causing event potentially unique.

General characterization of the biomechanical loading environment can, however, assist in developing a better understanding of the mechanisms of injury in question. In particular, the approach proposed here has a direct forensic value in the analysis of image based evidence, e.g., CCTV video footage, from which more accurate measures of velocity, location, and angle of impact might be obtained.

## Data Availability Statement

The datasets generated during and/or analyzed during the current study are not publicly available due to restrictions stipulated by the ethical approval for the study in order to protect patient confidentiality. The FEHM model used in this work is available on http://jerugroup.eng.ox.ac.uk/fehm.html and on the Oxford University Innovation Software Store https://process.innovation.ox.ac.uk/software. The ML pipeline is available under academic license on http://jerugroup.eng.ox.ac.uk/mltbi.html and on the Oxford University Innovation Software Store. Requests to access the datasets should be directed to antoine.jerusalem@eng.ox.ac.uk.

## Ethics Statement

The studies involving human participants were reviewed and approved by South central—Berkshire Research Ethics Committee. The patients/participants provided their written informed consent to participate in this study.

## Author Contributions

AS, NV, DG-G, MJ, J-MP, and AJ wrote the article. TL and NV designed the clinical programme. TL produced the clinical data. NV produced the functional clinical criterion and post-processed the clinical data. MJ produced the forensic analysis. DG-G produced the constitutive models. AS implemented the model and performed the simulations. J-MP and AJ designed the overall computational study. All authors contributed to the article and approved the submitted version.

## Conflict of Interest

JM-P was employed by the company Lurtis, Ltd. The remaining authors declare that the research was conducted in the absence of any commercial or financial relationships that could be construed as a potential conflict of interest.
